# Controlling patient participation during robot-assisted gait training

**DOI:** 10.1186/1743-0003-8-14

**Published:** 2011-03-23

**Authors:** Alexander Koenig, Ximena Omlin, Jeannine Bergmann, Lukas Zimmerli, Marc Bolliger, Friedemann Müller, Robert Riener

**Affiliations:** 1 Sensory-Motor Systems Lab, Department of Mechanical Engineering and Process Engineering, ETH Zurich, Switzerland; 2 Spinal Cord Injury Center, Balgrist University Hospital, University Zurich, Switzerland; 3 Hocoma AG., Volketswil, Switzerland; 4 Schön Klinik Bad Aibling, Germany

## Abstract

**Background:**

The overall goal of this paper was to investigate approaches to controlling active participation in stroke patients during robot-assisted gait therapy. Although active physical participation during gait rehabilitation after stroke was shown to improve therapy outcome, some patients can behave passively during rehabilitation, not maximally benefiting from the gait training. Up to now, there has not been an effective method for forcing patient activity to the desired level that would most benefit stroke patients with a broad variety of cognitive and biomechanical impairments.

**Methods:**

Patient activity was quantified in two ways: by heart rate (HR), a physiological parameter that reflected physical effort during body weight supported treadmill training, and by a weighted sum of the interaction torques (WIT) between robot and patient, recorded from hip and knee joints of both legs. We recorded data in three experiments, each with five stroke patients, and controlled HR and WIT to a desired temporal profile. Depending on the patient's cognitive capabilities, two different approaches were taken: either by allowing voluntary patient effort via visual instructions or by forcing the patient to vary physical effort by adapting the treadmill speed.

**Results:**

We successfully controlled patient activity quantified by WIT and by HR to a desired level. The setup was thereby individually adaptable to the specific cognitive and biomechanical needs of each patient.

**Conclusion:**

Based on the three successful approaches to controlling patient participation, we propose a metric which enables clinicians to select the best strategy for each patient, according to the patient's physical and cognitive capabilities. Our framework will enable therapists to challenge the patient to more activity by automatically controlling the patient effort to a desired level. We expect that the increase in activity will lead to improved rehabilitation outcome.

## 1 Introduction

Stroke is one of the most common causes of disability, affecting between 100 and 200 subjects per 100.000 citizens in the western world [1]. Treadmill training has been shown to be beneficial to regain walking functionality after stroke and has been established as gold standard in gait rehabilitation [2]. Robots such as the Lokomat [3-5], the Lopes [6], the Autoambulator http://www.healthsouth.com, the GaitTrainer [7] or the Walk Trainer [8] have become increasingly common in gait rehabilitation, as they allow for longer training duration and higher training intensity [9].

Despite an increasing amount of available gait robots, determination of their effectiveness has remained controversial. Some studies found robot-assisted therapy superior to manual therapy [10,11], while other studies drew the inverse conclusion [12-14].

Active contribution in a movement was shown to be crucial for motor learning and rehabilitation [15,16]. As gait robots are strong enough to move the patient's legs along a predefined walking trajectory, active participation of the patient can be seen as a key factor to improve the success of gait robots [17,18]. A lack of active participation might explain the inconclusive effect of rehabilitation robots, as subjects can behave passively in the robot as shown in studies from Israel et al. [18] and Hidler et al. [17], who found decreased muscle activity for robot-assisted walking compared to non assisted walking. On a biomechanical level, cooperative "assist-as needed" controllers can promote active participation [19]. On a cognitive level, visual feedback was shown to help patients to focus on their walking movement [20]. Virtual environments were shown to improve motivation of patients [21,22] and increased rehabilitation success [23].

However, there is no effective method for controlling patient participation during robot assisted gait training to a desired level. Due to the broad variety of physical and cognitive impairments of stroke patients, a "one-size fits all" solution for control of patient participation is unlikely to suit the demands of all patients. In particular, severe cognitive impairments limit the ability of the patient to understand which movements are recommended by the therapist and which (movements) are beneficial for therapeutic success.

In this paper, we present several approaches to controlling patient participation during robot-assisted gait therapy. HR and interaction forces between robot and patient were used as indicators of patient activity. We provide a metric that allows selecting the solution that best suits the patient's demands in terms of physical and cognitive impairment. With this approach, we expect an increase in activity during training compared to normal robot-assisted therapy which could have a beneficial effect on the rehabilitation outcome.

## 2 Methods

To be able to control patient participation during robot-assisted walking, it was necessary to define and quantify the amount of participation. Patient participation was quantified in two ways: by HR, a physiological parameter that reflected physical effort during body weight supported treadmill training [24] and by a weighted sum of the interaction torques (WIT) between robot and patient, recorded from hip and knee joints of both legs [20].

We introduced two different approaches to performing activity control that would suit various levels of physical as well as cognitive impairments of the patient. One approach was based on adaptation of treadmill speed during walking; the other was based on instructions given by visual information from a virtual environment. These two methods were experimentally evaluated using the Lokomat gait orthosis [3,5] in three experiments with five stroke patients each.

### 2.1 Definition of patient participation

The robot could be operated with varying degrees of supportive force, which significantly influenced patient participation. If the impedance controller was set stiff, the robot was position controlled. If the impedance was set low, the patient could lead the walking movement him or herself. At high assistive forces, the patient was able to push against the orthosis in direction of the walking movement, thereby overemphasizing the walking movement. Conversely, the patient could also behave passively and obtain a major contribution of the torques required for walking from the robot. The lower the impedance of the robot, the more torque the patient had to generate him or herself. At zero impedance, the robot did not provide any torque to assist the movement but behaved transparently by hiding its gravitational, coriolis and friction forces, as well as its inertia.

We defined patient activity during robot-assisted gait rehabilitation to be high when the patient actively contributed to the walking movement. The patient had to keep the assistive torque of the gait orthosis to a minimum and would perform the walking movement him or herself. At high impedance, the walking movement was fully prescribed by the gait robot. The patient was then able to perform active voluntary movements; pushing into the orthosis, the patient could overemphasize the walking movement and expended additional energy.

Conversely, patient activity was defined as low if the patient did not actively contribute to the walking movement. This was also only possible at high impedance settings, as the gait robot then provided most of the torque necessary to perform the walking movement and the patient was mostly moved by the gait robot in the walking trajectory. Gait speed, amount of body weight support and amount of assistive force generated by the orthoses influenced the effort necessary to perform the walking movement. The patient was forced to expend more energy during training when gait speed was increased, body weight support decreased or assistive force was decreased [24].

### 2.2 Quantifying patient participation

#### 2.2.1 Physiological quantification of patient participation

The electrocardiogram was recorded with a gTec http://www.gtec.at amplifier, sampled at 512 Hz, filtered with a 50 Hz notch filter and bandpassed with a 20-50 Hz Butterworth filter of 4^th ^order. HR was then extracted in real time using a custom steep slope detection algorithm adapted from [25]. All software was implemented in Matlab 2008b http://www.mathworks.com

#### 2.2.2 Biomechanical quantification of patient participation

**Figure 1:**
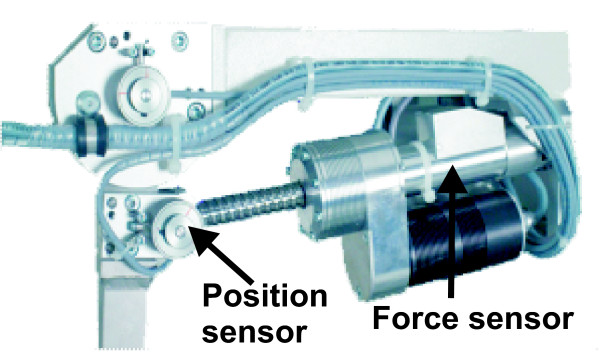
**Location of force and position sensors in the hip joint of the Lokomat gait orthosis (Image courtesy: Hocoma AG, Volkeswil, Switzerland)**.

To quantify physical effort from a biomechanical measure, we computed the WIT between robot and patient, recorded from hip and knee joints of both legs, using the standard Lokomat force sensors located in line with the linear guides (Figure 1).

For each step, the interaction torques of all four joints were computed from the force recordings, weighted using the weighting function of Banz et al. [20] and summed up. In previous work, Banz et al. 2008 had investigated whether the interaction torques could be used to distinguish a physiologically desired movement pattern that would be beneficial for rehabilitation outcome from a walking pattern that would not be desired, as rated by expert physiotherapists. The result was the weighting function that is used to compute the WIT values [20]. The WIT has a high positive value if the patient performs an active movement which is therapeutically desired and a negative value if the patient is passive or resists the walking pattern of the orthosis. Values around zero mean that the patient is able to minimize the interaction torques between his legs and the orthosis. Details on the computation and their physiotherapeutic interpretation can be found in [20,26,27].

The raw, un-weighted interaction torques between the patient and the orthosis could have been used to quantify, how much the patient contributed to the walking movement him or herself. However, raw torque exchange is not a suitable measure for patient activity, as therapeutically undesired movements can result in large interaction torques between Lokomat and human. Spasticity, for example, can cause large interaction torques, but usually does not contribute to a physiologically meaningful gait pattern.

### 2.3 Controlling patient activity with visual instructions

Patients that were cognitively capable of understanding virtual tasks and producing voluntary force were provided with real time feedback on their current activity using visual displays. With voluntary physical pushing effort, the patient had to match the current effort to a desired effort displayed on a screen. In this case, the control loop was closed via a visual feedback loop, as the instructions to the patient were given visually. The virtual stimulus was designed to be as easy and intuitive as possible such that patients with cognitive impairments were able to understand and perform the task. All action in the virtual environment took place on a straight path in the middle of the screen such that patients with partial neglect of the visual field could use the virtual environment.

**Figure 2:**
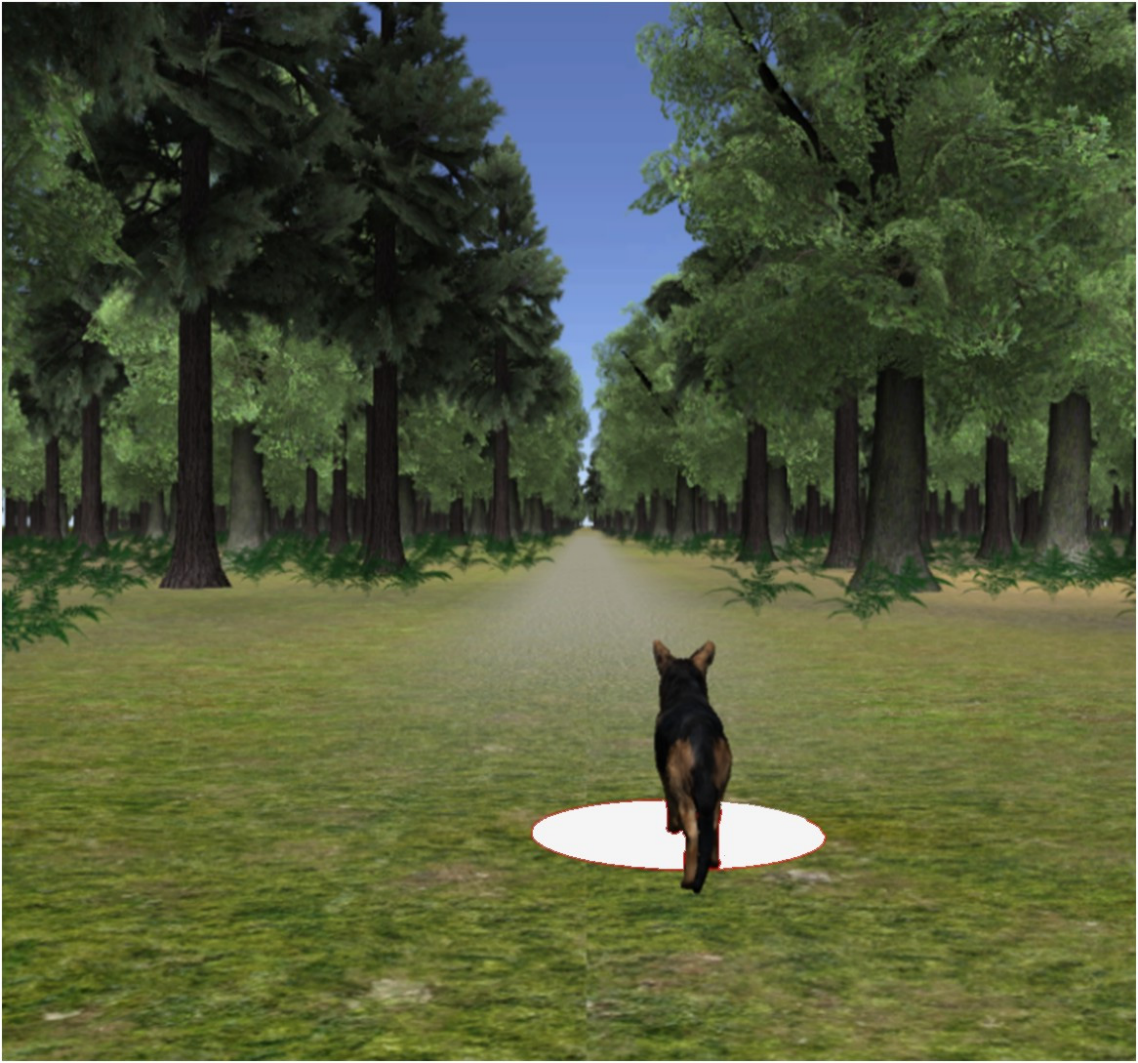
**Virtual scenario used for control of WIT and HR**. The distance between the dog (desired effort) and the white dot (actual effort) is the visual instruction to the patient. By increasing or decreasing his/her effort, the patient controlled the walking speed of the white dot.

The desired patient effort was displayed by the position of a dog walking in a virtual forest scenario (Figure 2). The current patient effort was displayed as a white dot on the floor of the virtual scenario. By increasing effort, the white dot moved faster, by decreasing effort, the white dot moved forward slower. The patients were instructed to place the white dot underneath the dog. This means the patients knew they had to increase their effort if the dog was walking too far ahead of the white dot and decrease their effort if the dog was walking behind the white dot. The distance between dog and virtual character displayed the difference between the desired and the actual effort of the patient.

**Figure 3:**
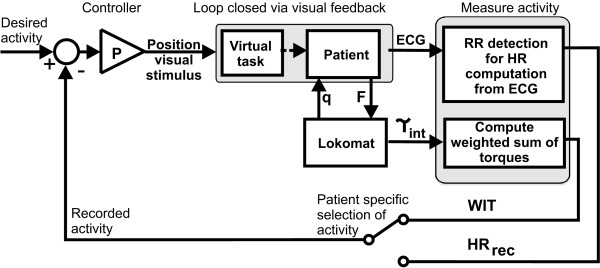
**Control scheme for control of activity via a visual stimulus**. Active participation is measured by HR or weighted interaction torques (WIT). The control loop is closed by the visual feedback to the patient. T_int _are the interaction torques between Lokomat and human. If HR control is chosen, mean HR is extracted in real time from the ECG and compared to a desired HR value. If WIT is controlled to a desired value, the current WIT values are computed from the interaction torques as detailed in the section 'Quantifying patient participation' and in Banz et al. [20]. The position of the visual stimulus is computed with a P gain.

Control of HR and WIT via visual stimuli was performed with the same stimulus for both measures of patient activity. The error between desired and recorded activity was mapped with a P gain to a distance between the virtual character and the dog (Figure 3).

### 2.4 Controlling HR using treadmill speed

Adaptation of treadmill speed allowed us to control patient activity to a desired temporal profile without the use of a virtual task. This would be necessary when the patient is cognitively not capable of understanding visual feedback, or physically not capable of exerting enough voluntary physical effort to control the virtual task. We imposed a higher physical load on the patient by increasing gait speed such that the patient was forced into a walking movement, which required increased activity. Conversely, lower gait speeds demanded less physical activity of the patient.

**Figure 4:**
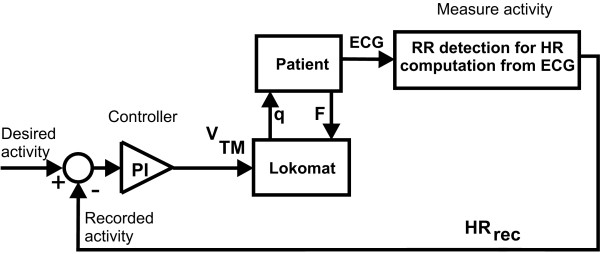
**Control scheme for HR control via treadmill speed**. T_int _are are the interaction torques between Lokomat and human. Mean HR is extracted in real time from the ECG and compared to a desired HR value. The error is fed into a PI controller that sets the gait speed of treadmill and Lokomat.

HR was controlled using a PI controller with anti-windup that adapted treadmill speed (Figure 4). PI control was chosen as it is well established in control systems design and has previously been used in HR control of healthy subjects. A discussion of advantages and disadvantages of previous approaches to HR control and their applicability in stroke subjects can be found in the Discussion section.

P and I controller gains were set to 0.05 and 0.01 respectively. The gains were tuned in pre-experiments using the Ziegler Nichols method [28], a standard methods for controller gain tuning when recorded data of a step input is available, and then fixed for all other subjects. Baseline HR was recorded at 1.5 km/h.

### 2.5 Experimental protocols

We performed three experiments (Table 1) and controlled HR via treadmill speed (experiment 1), via a visual stimulus (experiment 2) and WIT via the virtual task (experiment 3). Control of WIT was only performed using a virtual task, not via adaptation of treadmill speed. As described in the section "Quantifying patient participation", the patient had to actively participate in the walking movement to reach a high WIT value. The virtual task gave direct feedback on the current WIT and the patient could react to this visual feedback by increasing his or her activity voluntarily. Same was true for HR control via visual feedback. Treadmill speed as a control variable for HR was also used, as increased walking speed led to increased energy expenditure and therefore to increased HR. High WIT values, however, required the subject to voluntarily perform the walking movement in a therapeutically desired way, which was not controllable by treadmill speed alone.

**Table 1:** Overview over the experiments performed.

	Patient participation quantified by	
Control via	HR	WIT
Treadmill speed	Experiment 1	-
Visual Stimulus	Experiment 2	Experiment 3

Each experiment was performed with 5 stroke subjects

All three experiments were performed with 5 stroke patients, resulting in recordings of 15 patients (Table 2). The gait orthosis Lokomat [3,5] (Hocoma Inc., Volketswil, http://www.hocoma.com) was used for all experiments, but our approach is generalizable to any gait robot that is equipped with force sensors. In the experiments with virtual environments, subjects walked in the Lokomat at 2 km/h with maximal supportive force by the robot and individual body weight support settings determined by the therapist.

**Table 2:** Characteristics of patients of HR and WIT control experiments

	Pat No	Gender	Age [y]	Time since incident [m]	Lesion (side, infarction type)	β-blocker	FAC	Cognitive deficits
**HR control** **via treadmill speed**	1	m	43	29	r. hemorrhagic	no	3	Medium attention deficit
	2	w	52	5	l. ischemic	no	3	Medium attention deficit
	3	w	33	22	l. ischemic	no	2	n.a.
	4	m	49	29	l. hemorrhagic	no	2	Medium attention deficit
	5	m	57	23	r. ischemic	no	2	n.a.

**HR control** **via virtual stimuli**	6	m	36	5	r. ischemic	no	1	Small memory deficits
	7	m	71	2.5	r. ischemic	no	2	Neglect left
	8	m	68	2.5	r. ischemic	no	4	none
	9	m	55	3.5	r. hemorrhagic	no	0	Small attention deficit, neglect
	10	m	67	2	r. ischemic	no	3	Medium attention deficit, neglect left

**WIT control** **via visual stimuli**	11	m	65	1.5	l. ischemic	yes	5	Small attention deficits
	12	m	62	2	r. ischemic	yes	n.a.	None
	13	m	68	2.5	r. ischemic	no	4	None
	14	m	67	2	r. ischemic	no	3	Small attention deficits
	15	f	58	3.5	r. ischemic	no	n.a.	Small attention deficit, neglect left

Gender: m = male, f = female, l = left, r = right, FAC = Functional Ambulation Classification (0 = patient can only walk with the help of at least 2 people, 5 = patient is a communal walker), n.a. = data not available

During HR control via treadmill speed, the combination of the path control mode [19,29] with a modified Lokomat software allowed walking speeds up to 4 km/h. The maximal walking speed was determined for each patient, as not all patients were physically capable to walk at the maximal possible gait speed of 4 km/h. Minimum body weight support was identified for each patient individually by decreasing unloading at maximal walking speed in steps of one kilogram. Minimum body weight support was set right before the gait pattern degraded visibly as rated by the attending physiotherapist. The unloading was then kept constant over the whole training session. All patients of HR control experiments were instructed to refrain from coffee, nicotine, chocolate, black tea and energy drinks up to 4 hours prior to the experiment. HR control was only performed with patients that did not take beta blocking medication. All patients or their legal representative gave informed consent.

**Figure 5:**
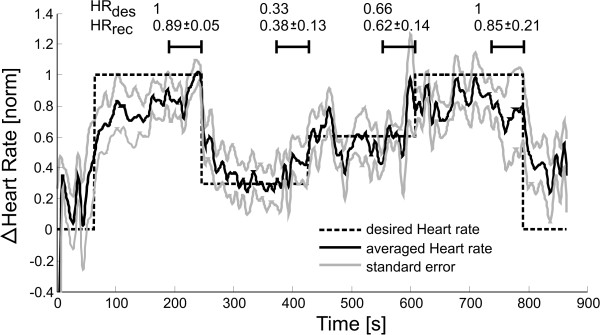
**Results of HR control via adaptation of treadmill speed**. Results were normalized and filtered with a zero-phase forward/backward low pass filter with cut-off frequency of 1 Hz to show the underlying trend.

**Figure 6:**
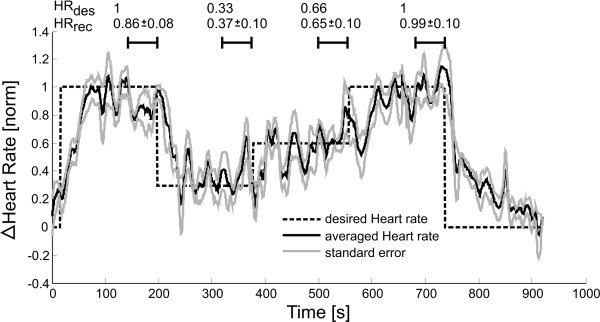
**Results of HR control via visual instructions**. Results were normalized filtered with a zero-phase forward/backward low pass filter with cut-off frequency of 1 Hz to show the underlying trend.

**Figure 7:**
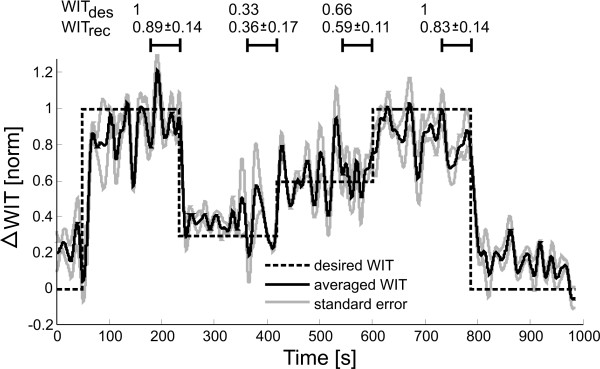
**Results of WIT **[20]**control via visual instructions**. Data was normalized and filtered with a zero-phase forward/backward low pass filter with cut-off frequency of 1 Hz lowpass to show underlying trend.

In all experiments, subjects were allowed to walk for ten minutes to get acquainted to the Lokomat. During these ten minutes, we also determined the baseline heart rate at a gait speed of 1.5 km/h. Lower gait speeds were reported to feel unnatural by the patients. If patients were walking in a virtual environment, they could also exercise the task during these ten minutes. We controlled HR or WIT to a desired temporal profile which included four distinct conditions of patient activity: low, intermediate, high and very high (100%, 33%, 66%, 100% as shown in Figure 5 Figure 6 and Figure 7 dashed line). Each condition was set to be three minutes long. Three minutes was a tradeoff between reaching steady state of HR and keeping the duration of the experiment sufficiently short such that the whole recording was kept below 30 minutes, which was requested by therapist thus avoiding overexertion of the patient.

The desired profile was scaled in amplitude to the maximal and minimal values of HR and WIT of each subject individually. In the virtual reality approach we identified patient specific limits of HR or WIT during the exercise time, by asking the subjects to perform at their respective maximal and minimal level of activity. In the treadmill speed approach, we identified the maximal HR before the experiment by letting the patient walk at his/her maximally tolerable walking speed.

### 2.6 Controller performance evaluation

Controller performance was evaluated by normalizing the recorded HR/WIT for each patient after his or her minimal and maximal HR/WIT. Data was then low pass filtered with a zero-phase Butterworth filter with cut-off frequency of 1 Hz to show the underlying trend. For heart rate data, the cut-off frequency of 1 Hz was experimentally determined to remove heart rate fluctuations caused by heart rate variability. We computed mean and standard error of HR and WIT, taken over the last minute of each condition to quantify steady state behavior rather than transient behavior. Statistical tests were used to compare the four desired conditions of physical effort (dashed lines in Figure 5, Figure 6 and Figure 7). In addition, we compared the three approaches to investigate, if the results of the three different approaches differed significantly from each other. Both tests were done with a Friedman test with Bonferroni correction. Significance level was set to 0.05 for all tests. Data processing was done using Matlab http://www.mathworks.com, statistical analysis was performed using IBM SPSS http://www.spss.com.

## 3 Results

### 3.1 Control of HR via treadmill speed

HR control of stroke patients via adaption of treadmill speed was performed successfully (Figure 5). Minimal and maximal HR values were used for normalization (summarized Table 3) such that we could compute the average tracking performance of the controller. The mean HR values of the last minute of each condition are summarized on the top of Figure 5. Patient 2 had to be excluded from the analysis, as he could not complete the desired protocol due to spasticity in the ankle joint of the affected leg caused by the physical effort of walking on the treadmill. All of the other subjects informally reported to be very exhausted at the end of the recording.

**Table 3:** Minimum and maximum HR values of each patient used for control of HR via treadmill speed and visual stimuli.

	Pat	Minimum HR	Maximum HR
HR control via treadmill speed	1	60	75
	2	97	107
	3	79	89
	4	80	92
	5	85	97

HR control via visual instructions	6	90	105
	7	75	90
	8	110	117
	9	93	103
	10	104	112

These values were used determined during the initial baseline recording and used for normalization during data processing. The desired HR profile to be tracked was scaled with these values to enable patient-specific exercise.

### 3.2 Control of HR via visual instructions

HR control of stroke patients was successfully performed via visual instructions from a virtual environment. As described in the methods section, subjects obtained instruction from the virtual environment to increase or decrease their voluntary physical effort and thereby their HR. Figure 6 shows the normalized increase in heart. The success of controlling HR with visual instructions was quantified by mean HR and standard error of all five subjects. The mean HR values of the last minute of each condition are summarized on the top of Figure 6.

It was necessary to adjust the baseline and maximal HR increase individually for each subject to provide patient-specific control of HR. In average, we were able to increase HR by 11 ± 4 bpm. Normalization values are summarized Table 3.

### 3.3 Control of WIT via visual stimuli

Control of WIT by means of a virtual stimulus was also performed successfully in five stroke patients. Tracking performance was quantified by mean WIT and standard error of all five subjects. The mean WIT values of the last minute of each condition are summarized on the top of Figure 7. It was necessary to adjust the baseline and maximal WIT increase individually for each subject to provide patient-specific control of WIT. Normalization values are summarized in Table 4.

**Table 4:** Minimum and maximum WIT values of each patient used for control of WIT via visual instructions.

Pat	Minimum WIT	Maximum WIT
11	0	90
12	-90	27
13	-27	81
14	-36	9
15	-45	0

These values were determined during the initial baseline recording and used for normalization during data processing. The desired WIT profile to be tracked was scaled with these values to enable patient-specific exercise.

While the levels of 30% and 60% of maximal WIT could be tracked well, subjects had problems in reaching maximal desired WIT. They could reach the desired maximal level for short time, but quickly became too exhausted to keep the effort at this level.

### 3.4 Statistical comparison between the three approaches

**Figure 8:**
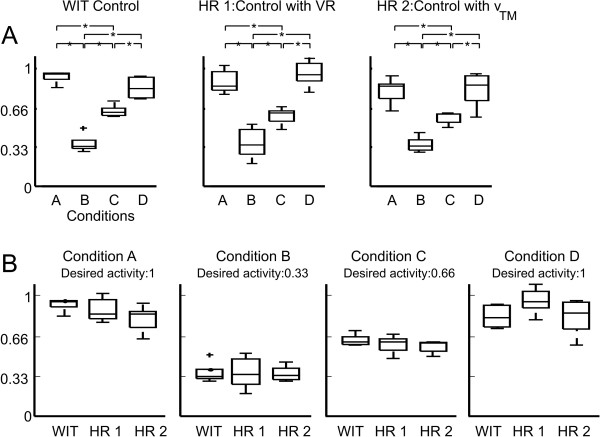
**Boxplots comparing the three different approaches**. WIT = WIT control with VR. HR1 = HR control with VR. HR2 = HR control with treadmill speed. Conditions A, B, C and D refer to the different levels of activity (100%, 33%, 66%, 100%). A: within one control approach, all conditions (except A compared to D) differ statistically. B: No significant differences were found between WIT, HR1 and HR2 for any condition.

The statistical analysis of each control approach showed that subjects could track the desired performance condition A-D (100%, 33%, 66%, 100% as shown in Figure 5, dashed line) with all three approaches (Figure 8 top). The comparison between the three different approaches showed that all approaches worked equally well for all conditions A-D (Figure 8 bottom). No significant differences were found between the different approaches.

## 4 Discussion

The overall goal of this paper was to investigate approaches to controlling active participation in stroke patients during robot-assisted gait therapy. We quantified patient effort in two ways: by HR and by a weighted sum of interaction torques (WIT - see methods section). For validation of our approach, we performed three experiments with stroke patients and controlled HR and WIT to a desired temporal profile.

Although active physical participation during gait rehabilitation was shown to be crucial for recovery from stroke [15], patients can behave passively during rehabilitation and therefore might not maximally benefit from the gait training. This might explain why several studies reported inconclusive results on the effects of robot-assisted gait therapy compared to manually-assisted gait therapy after stroke or spinal cord injury [14,30].

We successfully controlled patient participation to a desired level (Figure 5 Figure 6 and Figure 7). Depending on the patient's cognitive capabilities, this was either done by voluntary patient effort using visual instructions or by forcing the patient to varying physical effort by adapting the treadmill speed. In addition to adapting to the cognitive capacities of the patient, an initial magnitude scaling of the desired temporal control profile allowed adaptation to patient individual physical capabilities. Four levels of patient activity were targeted: 100%, 66%, 33% and again 100% of maximal participation (Figure 5 dashed line). Using three different approaches, all patients could equally well track the desired temporal profile, independent on their cognitive or motor impairments (Figure 8 top). Results showed no statistical differences in their applicability to patients (Figure 8 bottom). Our framework is intended to enable therapists to challenge the patient to active participation by automatically controlling the patient effort to a desired level.

### 4.1 Patient individual control of participation

One problem of controlling patient participation is the necessity of scaling the desired participation to a level, where the patient is able to perform at his or her individual capabilities.

The maximal and minimal WIT values (Table 4) reflect the individual physical ability of each patient. Although the WIT is a unit less quantity, healthy subjects could reach values between -400 while strongly resisting the orthosis movement during the walking pattern to +400 while maximally overemphasizing the walking movement and pushing into the orthosis. Patient 5 could only reach a maximal value of 0 which corresponds to the ability to perform the walking movement himself without being able to generate any additional pushing force in walking direction. Patient 1 on the other hand could reach a value of 90 which means that this patient was able to voluntarily push into the orthosis. Nevertheless, both patients could receive a challenging training that was adjusted to their individual capabilities.

During control of HR in experiment 1 and 2, the HR recorded at baseline reached from 60 bpm (Table 3 patient 1) to 110 bpm (Table 3 patient 8). With the limitations of gait speed imposed by the Lokomat and the physical abilities of the patients, some patients could only reach an increase in heart rate of 7 bpm (Table 3 patient 8), while others could be controlled in a range of 15 bpm. We could still provide challenging training sessions to the patients, independent on their individual physical capabilities, as all patients informally reported to be exhausted after HR control experiments,

### 4.2 A metric for patient individual control of physical activity

Based on the three successful approaches to controlling patient participation, we propose a metric which enables clinicians to select the best strategy for each patient, according to the patient's physical and cognitive capabilities. Controlling WIT requires the patient to have a cognitive understanding of a therapeutically desired gait pattern and the physical capability to alter the current gait pattern according to the performance feedback. We therefore consider WIT control to be the most challenging task that patients can perform.

HR on the other side will increase, as soon as the patient produces voluntary force against the position controlled orthosis, regardless if the movement is therapeutically beneficial or not. In order to control a virtual task with his/her HR, the patient needs to have the cognitive understanding of the task and must be able to produce physical effort. As this physical effort does not require the capability of the patient to adapt his or her gait pattern to a therapeutically desired pattern, the physical as well as the cognitive abilities of the patient do not have to be as intact as during WIT control. Patients with severe impairments might not be able to understand visual performance feedback or not be capable of generating enough pushing effort to increase their HR. For these patients, we propose HR control via adaptation of treadmill speed since higher gait speed in the Lokomat will increase HR regardless of the ability of the patient to voluntarily push into the orthosis.

While our metric will allow controlling participation in a wide range of patient groups, not all patient groups will benefit from it. Patients taking Beta blocking medication will not be able to exercise in HR control mode, as Beta blockers were shown to decrease HR variability and limit the adaptation of HR to physical stress [31]. These patients can still benefit from WIT control. Patients that are unable to produce directed, voluntary effort will neither be able to increase their HR by increased power expenditure, nor will they be able to control their WIT to a desired level. Furthermore, if the cognitive impairment does not allow the use of visual instructions and physical impairment prohibit walking at treadmill speeds that allow HR control via adaptation of treadmill speed, then control of patient participation will not be possible.

**Figure 9:**
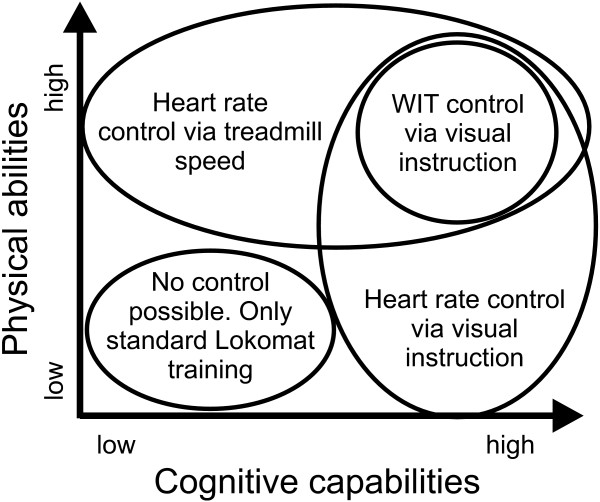
**Selection matrix for optimal training of stroke patients, depending on the patient's cognitive and physical impairments**. Mildly affected patients can exercise in all three modes: HR control via treadmill speed and visual instructions and WIT [20] control via visual instructions. Patients with strong cognitive deficits might only be able to exercise in HR control mode via treadmill speed. Patients that are capable of understanding a virtual task but are physically limited in their capabilities of controlling the WIT to a desired value can still exercise in HR control mode via visual instructions.

During the study design, we recruited 15 different subjects that had never experienced virtual reality feedback in the Lokomat for the three experiments. Due to this broad patient base we could investigate whether our methods worked with a variety of different impairments. The next step will be a larger study that can provide statistical evidence for the metric proposed in Figure 9. An objective rating for the cognitive impairments of subjects such as the mini-mental state estimation [32] will then also be collected.

### 4.3 Cardiovascular training after stroke

Our proposed method combines the advantages of virtual reality augmented gait training with the benefits of cardiovascular training. Non-ambulatory patients that use HR control during Lokomat walking are able to combine gait training with cardiovascular training. The benefits of cardiovascular training come at no extra cost to benefits of gait rehabilitation.

The use of virtual reality might increase the training efficacy of robot assisted gait therapy compared to training without virtual environments, as recently demonstrated by studies of Mirelman [23] and Bruetsch [22]. HR control via visual feedback has not been performed during robot assisted gait training before. However, oxygen uptake was controlled to a desired trajectory via volitional pushing effort during robot assisted gait training [33]. Subjects had to increase and decrease their effort (and thereby their energy expenditure) according to a visual display which coded the deviation from a desired oxygen uptake value.

Cardiovascular training, such as treadmill based HR control, was shown to be beneficial to stroke survivors during gait rehabilitation [34]. Depending on the degree of impairments caused by the lesion, this training has been performed either on treadmills for less severe cases or on stationary bicycles in severely affected patients. Particularly non-ambulatory patients were not able to exercise on treadmills, but had to use stationary bicycles instead, where the problems of coordination and balance during walking did not need to be taken into consideration.

To our best knowledge, there has been no study in which HR of stroke patients was controlled during treadmill walking. In healthy subjects, treadmill based HR control has been successfully demonstrated using PID or *H*_∞ _control [35-37]. In these studies, HR increases of 30 beats per minute (bpm) were demonstrated; we only reached an average HR increase of 12 bpm using treadmill speed as control signal. This seems to be a very small increase compared to the results obtained in healthy subjects. However, previous approaches to HR control of healthy subjects were performed at walking speeds starting at 3.6 km/h [35-37], which are not feasible for most patients. In our patient group, only one individual was able to walk at speeds higher than 3.6 km/h. Pennycott et al. [33] controlled oxygen uptake during Lokomat walking, however only in healthy subjects and with the drawback, that the method needed an initialization time for parameter identification, which would shorten the duration available for actual cardiovascular training in patients.

In addition to treadmill speed, the amount of body weight support would have an impact on the effort which patients have to expend during walking. Unloading was shown to alter HR at constant walking speeds [24]. We decided not to use body weight support as a control variable. Increased body weight support reduced the loading to be carried by the patient during gait. High loading of the patient during treadmill training was shown to be a key factor for rehabilitation success [38]. In order to maximize the quality of gait training, it was decided to set body weight support to a fixed, patient-specific minimal value.

### 4.4 Clinical applicability of patient activity control

Despite all the advantages of HR control, there are two major drawbacks compared to WIT control. First, patients have to refrain from consuming any substance that might influence HR such as beta blockers, coffee, nicotine or tea. Controlling patient behavior in this way might not be possible in a clinical setting. In addition, HR control requires an additional computer for recording of ECG, and additional time for attachment of the electrodes before the training. Also, carefully monitoring of the ECG signal quality is necessary, which might degrade over the time course of a training session due to sweating or artifacts induced by the body weight support harness. WIT in comparison does not require additional setup time, no additional sensors and will also work in patients that are under the influence of any of the above mentioned substances. At this time, WIT control seems to be more likely to find transfer into a standard clinical setting with patients that are cognitively capable of understanding a virtual task.

## 5 Conclusion and Outlook

We presented automated control strategies for patient activity over a broad variety of cognitive and physical impairments of patients. Besides stroke patients, our approach could also be applicable for cardiac insufficiency patients that need to perform cardiovascular interval training in a safe environment that can provide body weight support and supports the walking movement via an impedance controlled orthosis if necessary. Further experiments will need to be performed on larger patient populations to shine light on the question, if robot-assisted gait training can be further improved compared to manual training by controlling active patient participation.

## List of Abbreviations

Bpm: Beats per minute; HR: Heart rate; WIT: weighted sum of the interaction torques.

## Competing interests

LZ is employed by Hocoma AG, Volketswil, Switzerland, manufacturer of the Lokomat. However, Hocoma as a company was not involved in planning, writing, finalizing, or approving the publication of this paper. L.Z.'s contributions to this article were based upon his independent scientific motivation and his scientific backgrounds. In addition, L.Z.'s contributions to this article resulted from the long-term scientific collaborations and research partnerships among ETH Zurich, University Hospital Balgrist, and Hocoma.

## Authors' contributions

AK adapted the Lokomat control software, wrote the manuscript, performed recordings and data processing. XO co-authored the manuscript, performed recordings and data processing. JB performed recordings. LZ programmed the virtual environment. MB co-authored the manuscript and performed recordings. FM co-authored the manuscript and provided clinical advice on the design of the methodology. RR co-authored the manuscript and provided engineering advice on the methodology. All authors read and approved the final manuscript.
